# A Striking Greenish-Yellow Endoscopic Appearance of Opportunistic Fungal Duodenitis in a Non-Human Immunodeficiency Virus (HIV) Immunocompromised Patient

**DOI:** 10.7759/cureus.111515

**Published:** 2026-06-25

**Authors:** Greco Gaspareantonio, Omar Ajhar, Zachary Neubert

**Affiliations:** 1 Gastroenterology, Halifax Medical Center, Daytona Beach, USA

**Keywords:** cryptococcus spp, duodenitis, immunosuppression, “opportunistic fungal infection”, renal transplant recipient, upper endoscopy

## Abstract

Opportunistic gastrointestinal infections in immunocompromised patients can present with atypical and misleading findings. We report a renal transplant recipient on chronic immunosuppression who presented with persistent nausea, vomiting, and abdominal pain. Endoscopy revealed a striking diffuse greenish-yellow discoloration of the duodenal mucosa with friability and superficial sloughing. Histopathology demonstrated Candida species and encapsulated yeast forms suspicious for Cryptococcus despite negative serum cryptococcal antigen testing. This case highlights a potentially underrecognized endoscopic phenotype of fungal duodenitis and underscores the importance of repeat endoscopic evaluation with targeted biopsy in immunocompromised patients when initial diagnostic studies are inconclusive.

## Introduction

Renal transplant recipients are at heightened risk for opportunistic infections due to chronic immunosuppressive regimens that impair systemic and mucosal immune defenses [1, 2]. Gastrointestinal fungal infections, while uncommon, can lead to severe morbidity and mortality, especially when diagnosis is delayed due to atypical clinical and endoscopic presentations [3, 4]. Typical endoscopic findings in duodenal injury usually include erythema, ulceration, or mucosal pallor; however, distinctive pigmentary changes are rarely reported [5-7]. Candida species are frequent fungal pathogens in immunocompromised hosts, and their intestinal expansion is associated with disruption of the bacterial microbiome, facilitating co-infections such as Clostridioides difficile [8, 9]. Cryptococcal gastrointestinal involvement is exceedingly rare and typically described in advanced human immunodeficiency virus (HIV) infection, with serum cryptococcal antigen assays showing limited sensitivity in localized disease [10-12]. Not only is Cryptococcosis gastrointestinal (GI) involvement rare, but its involvement in the duodenal has only been recorded in seven cases ever, with all of them being in advanced HIV/ acquired immunodeficiency syndrome (AIDS) patients [10, 11].

This report presents a case of localized fungal duodenitis with a striking greenish-yellow mucosal discoloration in a renal transplant recipient with profound pancytopenia, illustrating the diagnostic complexity and therapeutic challenges associated with opportunistic gastrointestinal infections in severely immunocompromised patients.

## Case presentation

A 59-year-old male presented with persistent nausea, vomiting, abdominal pain, and poor oral intake. The patient’s medical history was significant for end-stage renal disease status post renal transplantation in 2023, chronic immunosuppression with cyclosporine and prednisone, chronic pancytopenia, duodenal ulcer disease, recurrent cytomegalovirus (CMV) viremia, and prior Clostridioides difficile infection.

The patient presented on multiple occasions with intractable nausea and vomiting, resulting in acute pre-renal kidney injury, with serum creatinine ranging from 2.7 to 2.9 mg/dL. Complete blood count demonstrated persistent pancytopenia, with white blood cell counts ranging from 2.0 to 3.6 ×10³/µL, hemoglobin levels between 7 and 8 g/dL, and platelet counts ranging from 7 to 36 ×10³/µL.

Cross-sectional imaging revealed thickening of the duodenal wall with surrounding inflammatory changes [13, 14], mild hydronephrosis of the transplanted kidney, and a 1.5 cm pulmonary nodule in the left lower lobe. Initial esophagogastroduodenoscopy (EGD) demonstrated duodenitis; however, tissue sampling was inadequate for definitive diagnosis.

**Figure 1:**
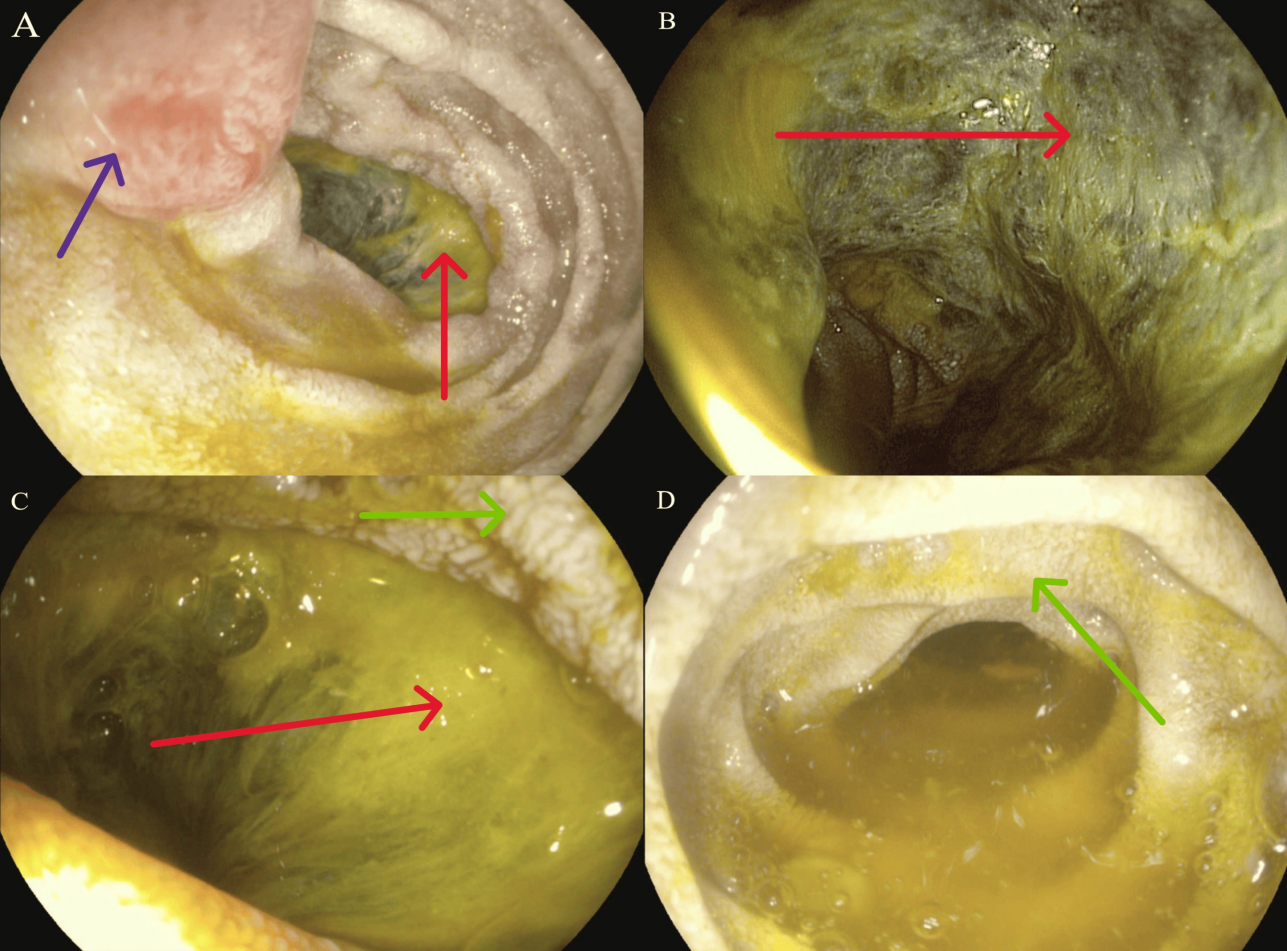
Endoscopic Findings (A) Endoscopic view of the duodenal mucosa at the level of the ampulla (purple arrow), beginning of the thick greenish plaque(red arrow) . (B, C) Diffuse greenish‑yellow duodenal mucosal plaque (red arrows), Duodenal Mucosa(green arrow). (D) Duodenal mucosa distal to the plaque (green arrow).

A repeat EGD revealed diffuse greenish-yellow discoloration of the duodenal mucosa with marked friability, scattered mucosal debris, and superficial sloughing (Figure 1). These endoscopic findings represented a visually distinct pattern not characteristic of typical inflammatory or ulcerative duodenal disease. Associated duodenal wall thickening was also noted.

Histopathologic examination of duodenal biopsies demonstrated fungal elements, including Candida species and encapsulated yeast forms highly suspicious for Cryptococcus. Alternative diagnostic considerations included severe inflammatory duodenitis, ischemic injury, medication-related mucosal injury, and other opportunistic infectious etiologies such as Histoplasma capsulatum or Blastomyces. However, the identification of fungal elements on histopathology, together with the patient's profound immunosuppression and persistent symptoms, supported an opportunistic fungal process as the leading diagnosis.

Given the patient’s persistent pancytopenia, a bone marrow biopsy was performed to evaluate for myelodysplastic syndrome versus medication-induced marrow suppression. Management required coordinated adjustment of antimicrobial therapy, modification of immunosuppressive medications, and supportive care measures.

The patient was ultimately discharged on a tailored antimicrobial regimen with close outpatient follow-up. 

## Discussion

This case describes a renal transplant recipient with chronic immunosuppression who developed localized opportunistic fungal duodenitis associated with a striking and unusual endoscopic appearance.

Novel endoscopic phenotype

The defining feature of this case was a diffuse greenish-yellow discoloration of the duodenal mucosa accompanied by friability and superficial sloughing. This presents a distinct diagnostic challenge, necessitating a comprehensive differential diagnosis that encompasses pseudomelanosis duodeni, systemic hemosiderosis, metastatic malignant melanoma, medication-induced pigmentation, and brown bowel syndrome.

In previously reported cases of infectious or inflammatory duodenal disease, endoscopic findings have most often consisted of mucosal erythema, ulceration, granularity, pallor, or plaque-like lesions rather than diffuse chromatic discoloration [4, 5, 10, 11]. Opportunistic gastrointestinal infections, including those caused by fungal and viral pathogens, are generally described as producing nonspecific inflammatory or ulcerative patterns [3-5]. A uniform greenish-yellow mucosal appearance has not been well characterized in the existing literature. This distinction supports the possibility that the observed chromatic change represents a visually recognizable but underreported manifestation of fungal duodenitis in immunocompromised hosts.

To our knowledge, this distinctive diffuse chromatic phenotype represents a previously underrecognized endoscopic manifestation of fungal duodenitis, with potential value as an early visual diagnostic clue in immunocompromised patients.

Diagnostic pitfall: negative serum testing

Histopathologic evaluation revealed Candida species and encapsulated yeast forms suspicious for Cryptococcus despite negative serum cryptococcal antigen (CrAg) testing. Although Cryptococcus most commonly presents with pulmonary or central nervous system involvement, gastrointestinal disease is an uncommon clinical presentation. In one autopsy series of disseminated or pulmonary cryptococcosis, gastrointestinal involvement was identified in 33% of cases [13]; however, this finding was typically incidental and not clinically apparent. Isolated duodenal involvement is exceedingly rare, with only seven cases reported in the literature before 2015, the majority occurring in patients with advanced human immunodeficiency virus infection [10, 11].

The discordance between tissue findings and negative serum CrAg highlights an important diagnostic limitation. Serum antigen assays may demonstrate reduced sensitivity in localized or low-burden disease, particularly in HIV-negative or non-critically ill patients in whom infection may remain confined to a single organ system. Prior studies have demonstrated reduced sensitivity of serum CrAg testing in HIV-negative patients, with sensitivity reported as low as 23.5% in cases of localized pulmonary disease using latex agglutination assays, compared to higher sensitivity in disseminated infection [15].

Accordingly, negative serum testing does not exclude cryptococcal infection in immunocompromised patients, and reliance on serum diagnostics alone may delay diagnosis. This limitation is particularly relevant given that most reported cases of gastrointestinal cryptococcosis have occurred in patients with advanced HIV infection, in whom disease burden is typically higher and diagnostic yield from serum testing is greater [16, 17]. These findings underscore the importance of repeat endoscopic evaluation with targeted biopsy in patients with persistent symptoms and atypical mucosal findings.

Pathophysiologic considerations

Renal transplant recipients are uniquely predisposed to opportunistic gastrointestinal infections as a result of impaired systemic and mucosal immune defenses [18, 19]. In this case, the concurrent identification of Candida species, suspected cryptococcal organisms, and prior Clostridioides difficile infection suggests that the observed mucosal injury likely reflects combined opportunistic infection in the setting of profound immune dysfunction.

Fungal overgrowth has been associated with disruption of the intestinal microbiome and may increase susceptibility to additional pathogens [20]. Furthermore, fungal colonization may alter the mucosal microenvironment and has been proposed to potentiate the virulence of co-infecting organisms, offering a plausible mechanistic explanation for the atypical macroscopic appearance observed in this case.

Hematologic considerations

The patient’s persistent multilineage cytopenias raised concern for an underlying bone marrow disorder rather than isolated medication-related toxicity. Although medications commonly used in transplant recipients are known contributors to cytopenias, the progressive and sustained nature of this patient’s abnormalities, in conjunction with peripheral smear findings, warranted further evaluation with bone marrow biopsy.

Post-transplant myelodysplastic syndromes and other marrow disorders are increasingly recognized. Large registry studies have demonstrated a significantly elevated incidence of myelodysplastic syndrome following solid organ transplantation. These conditions carry substantial infectious risk, with infection representing a leading cause of morbidity and mortality in affected patients and accounting for up to 64% of deaths in some series [21, 22].

Clinical management challenges

Management was complicated by concurrent infectious, renal, and hematologic abnormalities requiring multidisciplinary coordination among infectious disease, nephrology, hematology, and transplant services. Immunosuppressive therapy was adjusted by withholding mycophenolate while continuing cyclosporine and prednisone to preserve allograft function. The patient initially received broad-spectrum antimicrobial therapy, including oral vancomycin, daptomycin, and meropenem, for suspected infectious complications in the setting of profound immunosuppression and persistent cytopenias. Following repeat endoscopy and histopathologic identification of fungal organisms within the duodenal mucosa, antifungal therapy was initiated with anidulafungin, and fluconazole was subsequently added after cultures grew Candida albicans/dubliniensis. Daptomycin was later discontinued because of elevated creatine phosphokinase levels. Concurrent severe pancytopenia necessitated bone marrow biopsy and transfusion support during hospitalization. The patient demonstrated clinical improvement and was ultimately discharged with ongoing antifungal therapy and close outpatient follow-up with infectious disease, nephrology, and hematology specialists.

The incidental identification of a pulmonary nodule introduced additional diagnostic uncertainty, raising concern for disseminated fungal infection or malignancy in the setting of possible marrow dysfunction. These competing clinical priorities highlight the complexity of managing immunocompromised patients with multifactorial disease processes.

Clinical implications

Several important clinical lessons emerge from this case. Gastrointestinal symptoms in immunocompromised patients warrant persistent investigation, and nondiagnostic initial evaluations should not preclude repeat endoscopic assessment when clinical suspicion remains high.

Recognition of atypical endoscopic findings, such as diffuse chromatic mucosal changes, may provide an early visual clue to underlying opportunistic infection. Additionally, cytopenias in transplant recipients should not be attributed solely to medication effects without thorough hematologic evaluation.

Ultimately, immunosuppressed patients frequently present with competing risks, including infection, rejection, medication toxicity, and marrow dysfunction, necessitating a multidisciplinary approach to optimize diagnostic accuracy and clinical outcomes [23, 24].

## Conclusions

This case illustrates the profound diagnostic complexity involved in managing an immunocompromised renal transplant recipient presenting with a unique, diffuse greenish-yellow endoscopic phenotype of fungal duodenitis. The stark discordance between our tissue-proven histopathology and a negative serum cryptococcal antigen test underscores a critical clinical pitfall, demonstrating that serum diagnostics can fail in localized, low-burden gastrointestinal disease. Ultimately, when managing vulnerable patient populations with persistent symptoms, clinicians should not rely solely on systemic serologies or initial inconclusive studies.

This case strongly supports the necessity of performing repeat endoscopic evaluations with targeted, high-quality tissue biopsies to unmask atypical opportunistic pathogens. Recognizing these distinct mucosal color changes can serve as an early visual diagnostic clue, driving timely multidisciplinary intervention to optimize clinical outcomes and preserve allograft function.
